# Antibacterial activities, proposed mode of action and cytotoxicity of leaf extracts from *Triumfetta welwitschii* against *Pseudomonas aeruginosa*

**DOI:** 10.1186/s12906-019-2713-3

**Published:** 2019-11-19

**Authors:** Molly Mombeshora, Stanley Mukanganyama

**Affiliations:** Biomolecular Interactions Analyses Group, Department of Biochemistry, University of Zimbabwe, P.O. Box MP 167, Mount Pleasant, Harare, Zimbabwe

**Keywords:** *Triumfetta welwitschii*, Antibacterial, Toxicity, Haemolytic

## Abstract

**Background:**

*Pseudomonas aeruginosa* has become a main cause of Gram-negative infection, particularly in patients with compromised immunity. High rates of resistance to antibiotics are associated with nosocomial infections caused by *P. aeruginosa* strains. The search for novel antimicrobials has been necessitated by the emergence of antimicrobial resistance in some bacteria Plant-based antimicrobials has great potential to combat microbial infections using a variety of mechanisms. *Triumfetta welwitschii* plant roots are traditionally used to treat symptoms of diarrhoea and fever, suggesting that it possess antimicrobial and immunomodulatory effects. Since research investigating antimicrobial properties of the roots of *Triumfetta welwitschii* has been explored, there is need to investigate the antimicrobial activity of its leaf extracts in order to probe their prospective use as new antimicrobial agents that can be used to combat nosocomial infections. The objective of this study was to evaluate the antibacterial activities, the mode of action and cytotoxicity of *T*. *welwitschii* leaf extracts.

**Method:**

Extracts of *T*. *welwitschii* leaves were obtained using eight different solvents, the serial exhaustive extraction method and the cold maceration technique. In vitro antibacterial activity evaluation of the extracts was done on eight bacterial isolates using the broth microdilution method. The mode of action for the most potent extracts was investigated using the rhodamine 6G efflux assay and the propidium iodide-based membrane damage assay. Toxicity of the extracts was evaluated using the haemolytic and MTT (3-(4, 5-dimethylthiazol-2-yl)-2, 5-diphenyltetrazolium bromide) assays.

**Results:**

The results showed that acetone, ethanol and dichlorometane: methanol extracts had the most potent antibacterial activities against *Pseudomonas aeruginosa* (ATCC 27853). All three extracts caused membrane disruption of *P. aeruginosa* as shown by nucleic acid leakage. All three extracts were unable to inhibit efflux pumps.

**Conclusion:**

The presence of antibacterial activities and low toxicity shown by the extracts indicates that the plant may be a source of effective antibacterial against some bacterial infections caused by *P. aeruginosa*. The disruption of membrane integrity is one possible mode of action of antibacterial activity of the potent extracts.

## Background

Treatment of infectious diseases is becoming more challenging due to the development of resistance to multiple classes of antibiotics by bacteria. This is especially true for infections caused by *Pseudomonas aeruginosa*. *P. aeruginosa* is a frequent causative pathogen in nosocomial infections. The Gram-negative bacterium is associated with nosocomial pneumonia, and is frequently implicated in hospital-acquired bloodstream and urinary tract infections [1]. In an attempt to counteract resistance to antibiotics, a number of studies now focus on the search for new antimicrobials. Plants are one of the main targeted sources in the search for novel antimicrobials.

Constituents of plant origin provide a good source of antimicrobial compounds [2, 3], as plants have evolved a variety of diverse chemical strategies to combat attack from pathogens. The secondary metabolites of medicinal importance include alkaloids, flavonoids, tannins, terpenes, and phenolic compounds. These active constituents possess effective pharmacological activity [4]. *Triumfetta* w*elwitschii* Mast. belonging to the Tilicea family is an important medicinal plant largely used in the Southern African countries as traditional medicine. Its roots are crushed and used in the form of decoction to treat symptoms of diarrhoea [5]. A mixture of milk and roots of *T*. *welwitschii* is used as an oral antipyretic agent [6]. Root extracts of *T. welwitschii* has been reported to possess antiplasmodial activity [7] and antiproliferative activity against Jurkat cells [8]. Antibacterial activity against *Escherichia coli*, *Bacillus cerus* [9] and antimycobacterial activity against *Mycobaterium aurum* and *Myocobacterium smegmatis* has been reported from root extracts of *T*. *welwitschii* [10]. The current study shifts from investigating antimicrobial activity of the roots and focuses on the leaves of *T*. *welwitschii*. The leaves from the same family (Tilicea) of plants have been reported to possess analgesic and antimicrobial activity [11–13], indicating the potential for antimicrobial activity in leaves of *T*. *welwitschii*. The main classes of secondary metabolites found in *T*. *welwitschii* are flavonoids, phenols and coumarins (unpublished data from BIA laboratory). The primary objective of the current study was to investigate the antibacterial properties of the leaf extracts of *T*. *welwitschii* against six of some of the common nosocomial pathogens [14]. The secondary objectives were to evaluate the possible mode of action and cytotoxicity of the crude extracts.

## Methods

### Collection of plant material

*T*. *welwitschii* leaves voucher number C16 E7 were procured between January and April of 2017 from the communal lands of Centenary (16.8^o^S, 31.1167°E, and 1156 m above sea level), in the Mashonaland Central Province of Zimbabwe. The identification and authentication of the plant sample was done by Mr. Christopher Chapano of the National Botanical and Herbarium Garden (Harare, Zimbabwe). Permission to use the plant samples was granted by the Faculty of Higher Degrees Committee, Harare, Zimbabwe (HD/71/16). The leaves were washed with tap water several times to remove any soil or dust particles. Drying of the leaves was carried out under shade for 21 days.

### Preparation of extracts

All solvents used for extraction were of analytical grade and were obtained from Sigma Aldrich (Steinheim, Germany). The leaves were ground to a fine homogenous powder using a pestle and mortar. A total of 384 g of powder was obtained and stored. A mass of 50 g powder was placed in a plastic beaker and 500 ml of 50: 50 v/v dichloromethane (DCM): methanol added to the powder. The cold maceration method with modifications was used to extract phytochemicals from the powdered leaves [15]. Maceration involved soaking plant materials with a solvent in a beaker covered with foil paper. The mixture was allowed to stand at room temperature for a period 2 days with frequent agitation. Solvents of different polarities namely: hexane, DCM, acetone, ethyl acetate, methanol, ethanol and water were used to serially extract phytochemicals from a new powder sample. Serial exhaustive extraction [16] involved extracting sequentially with a non-polar solvent (hexane) to a moderately polar and finally polar solvent (water). The slurry obtained was filtered through a No. 1 Whatman filter paper. The filtrate obtained was concentrated under a vacuum using a rotary evaporator RII (BUCHI, LabortechnikAG, Switzerland). The extracts were dried to a constant mass under a fan in a fume hood cabinet. All extracts were stored in sterile tubes at − 4 °C until use.

### Chemicals used in assays

Chemicals used in the study included; ampicillin (A9518), levofloxacin (28266), dimethyl sulphoxide (DMSO) (D5879), thiazolyl blue (M2128), reserpine (R0875), sodium citrate (1613859), potassium ferricyanide (702587), sodium carbonate (1613757), glucose (G8270) and rhodamine 6G (R6G) (252433) were purchased from Sigma Aldrich (Germany). Tryptic soy broth (TSB) (22092), tryptic soy agar (TSA) (22091) and Roswell Park Memorial Institute media (RPMI) (8758) were also from Sigma Aldrich (Germany).

### Microbial strains and culture media

Six of some of the common nosocomial pathogens [14] were chosen for this study. Isolates of *Bacillus subtilis*, *Staphylococcus aureus*, *Pseudomonas aeruginosa*, *Streptococcus pneumoniae*, *Streptococcus pyogenes* and *Klebsiella pneumoniae* isolated from patients were supplied by Parirenyatwa Group of Hospitals (Department of Medical Microbiology, College of Health Sciences, Harare, Zimbabwe). *S. aureus* was isolated from an ear infection and *P. aeruginosa* from a urinary tract infection. Types of infection from which the isolation of *B. subtilis*, *S. pneumoniae*, *S. pyogenes* and *K. pneumoniae* were not specified. *P. aeruginosa* ATCC 27853 and *S. aureus* ATCC 9144 were acquired from the Microbiological Section in the Department of Biological Sciences at the University of Botswana (Gaborone, Botswana). Bacteria were kept as glycerol stocks at − 35 °C. For each assay bacteria were grown on tryptic soy agar (TSA) for 24 h at 37 °C, followed by inoculation in tryptic soy broth (TSB). Inoculum concentration was adjusted to10^6^ c.f.u/ml by diluting the inoculum using TSB using 0.5 McFarland standard.

### Determination of antibacterial activities of leaf extracts isolated from *Triumfetta welwitschii*

Antibacterial activities of the DCM: methanol, hexane, DCM, ethyl acetate, acetone, ethanol, methanol and water extracts were determined by reconstituting each extract in dimethyl sulfoxide (DMSO). Required concentrations (12.5, 25, 50 and 100 μg/ml) of the extracts were obtained by diluting using TSB. The broth microdilution method [17] with minor modifications was used to determine the effects of the extracts against ATCC strains of *P. aeruginosa* and *S. aureus*. Susceptibility of the clinical strains of *P. aeruginosa* and *S. aureus, K. pneumoniae*, *S. pneumoniae*, *S. pyogenes* and *B. subtilis* were also determined. Liquid cultures of each bacterium were grown in TSB media. These were diluted in fresh TSB and 100 μL was applied to the wells of a 96-well plate. In each case, approximately 2 × 10^6^ cfu/ml of exponentially growing cells was inoculated for each strain. The extracts or antibiotics (100 μL) were added to these wells in decreasing concentrations and mixed by pipetting. Cells in tryptic soy broth were used as the positive control. While cells exposed to the standard antibiotic were used as the negative control. Cell density of the plate was measured at 590 nm using a microplate reader (Tecan Genios-Pro microplate reader, Grödig, Austria) before incubation. Plate was incubated at 37 °C for 24 h, and cell density was measured. Growth of cells was determined by finding the difference of the pre-incubation value from the post-incubation value. Data are presented as percentage inhibition of inoculum. Percentage inhibition was obtained using the equation:

1 $$ \mathrm{Percentage}\ \mathrm{inhibition}=\frac{\left(\mathrm{positive}\ \mathrm{control}\ \mathrm{value}-\mathrm{sample}\ \mathrm{value}\right)\kern.5em \times 100}{\mathrm{positive}\ \mathrm{control}\ \mathrm{value}} $$

Ampicillin (0 to100 μg/ml) was used as the standard antibiotic used against *P. aeruginosa* and *S. aureus*. Ciprofloxacin (0 to1 μg/ml) against *S. pyogenes* and *B. subtilis*. Levofloxacin (0 to 1 μg/ml) was used against *K. pneumoniae*, *S. pneumoniae*.

### Determination of the possible mode of action of antibacterial

#### Membrane damage potential

The cell membrane damage potential of the DCM/methanol, acetone and ethanol extracts from leaves of *T*. *welwitschii* against the ATCC strain of *P. aeruginosa* was determined using propidium iodide as described by Moyo and Mukanganyama [9], with modifications. Propidium iodide is a dye capable of binding to nucleic acids of non-viable cells with damaged membranes only [18]. The dye is unable to enter viable cells, thus, it is useful for determining the effects of plant extracts on bacterial membranes. *P. aeruginosa* cells were grown by pipetting 200 μl of overnight inoculum into 200 ml TSB and incubating overnight at 37 °C with shaking in an incubator. The optical density of the cells was adjusted to an OD_600_ = 1.5 equivalent to 2 × 10^9^ c.f.u/ml using PBS. Cell suspensions were exposed to different concentrations of the extracts of a final concentration of 50 μg/ml, 100 μg/ml and 200 μg/ml for 30 min at 37 °C with shaking in an incubator. The negative control contained cells with no extract added. All test samples were prepared in triplicate. After incubation, 1 ml of each test sample was centrifuged at 11000 rpm. The pellet was washed with saline solution, resuspended in PBS and propidium iodide of a final concentration of 10 μg/ml added to the suspension. The mixture was kept in the dark for 10 min after which 200 μl of test samples were transferred to a 96-well plate. Fluorescence was measured at 544 nm Excitation and 612 Emission using an *f*_max_ spectrofluorometer (Molecular Devices, Sunnyvale, USA).

#### Determination of the extracts on drug transport activity

The transport of R6G dye out of cells as described by Chitemerere and Mukanganyam [19] was used to evaluate the effects of the acetone, ethanol and DCM/methanol leaf extracts as potential efflux pump inhibitors. Duplicate standards of R6G (0 μM to 3 μM) were prepared in PBS and their absorbance values determined at 527 nm using a Shimadzu UV/VIS UV-1601spectrophotometer (Shimadzu, Kyoto, Japan). A calibration curve was generated from values of absorbance obtained as a function of concentration using Graphpad™ version 5 for Windows (Graphpad™ Software Inc., San Diego, California, USA).

A sub-inhibitory concentration (25 μg/ml) of each extract was used in the R6G efflux assay using the laboratory strain of *P. aeruginosa* cells. The R6G efflux assay was carried out by growing 200 μL of an overnight culture of cells in three 200 ml nutrient broth in 2 L flasks and incubated overnight at 37 °C with shaking (120 r.p.m). Cells were centrifuged using a Rotafix 32A centrifuge (Hettich, Benin, Germany) and washed using phosphate buffer solution (PBS pH 7.4). Cells were resuspended in PBS containing sodium azide to a final concentration of 40 mg/ml. A final concentration of 10 μM R6G was added and the mixture incubated at 37 °C for an hour with shaking (120 r.p.m). Cells were collected by centrifuging at 4000 r.p.m. for 15 mins and cells exposed to the following reagents in six separate tubes containing: glucose, no glucose, glucose + reserpine, glucose + acetone leaf extract, glucose + ethanol leaf extract, glucose + DCM/methanol leaf extract. The final concentration of reserpine used was 80 μg/ml.

All samples were incubated at 37 °C for 1 h. Cells were collected by centrifugation at 4000 r.p.m. for 15 min and the supernatant was used for R6G efflux quantification. Optical density values of the R6G pumped out of the cells was determined using a Shimadzu UV/VIS UV1601 spectrophotometer (Shimadzu Corporation, Kyoto, Japan) at a wavelength of 527 nm. The calibration curve was used to interpolate concentrations of R6G in samples in the efflux assay based on their absorbance values.

### Evaluation of the toxicity of the leaf extracts

#### Determination of toxicity using sheep erythrocytes

The cytotoxicity effects of the DCM/methanol, acetone and ethanol extracts from leaves of *T. welwitschii* against erythrocytes from sheep was determined using the haemolysis assay as described by Malagoli [20], with modifications. A volume of 50 ml sheep blood was collected and added to an equal volume of Alsever solution. Blood was centrifuged at 3000 r.p.m. for 10 min and the supernatant was discarded. The residue was washed three times with a 1:5 volume of PBS. The resulting cells were diluted four-fold using PBS to give an erythrocyte suspension. Extracts were prepared in PBS and final concentrations of 50 μg/ml (^1^/_2_MIC), 100 μg/ml (MIC) and 200 μg/ml (2MIC) were used in the assay. The erythrocyte suspension (500 μl) was mixed with 500 μl test sample extract and incubated for 90 min at 37 °C. All test samples were prepared in triplicate. After incubation, the tubes were spurn at 3000 r.p.m. for 1 min in a microcentrifuge (Geratebau Eppendorf GmbH, Engelsdorf, Germany). The positive control with 100% haemolysis was obtained by mixing 200 μl erythrocyte suspension with 1.5 ml Drabkin’s reagent; the negative control was a mixture of 500 μl erythrocyte suspension and 500 μl PBS. Aliquots of 200 μl of supernatant were transferred into 96-well plates. The absorbance (Abs) of haemoglobin released was measured at 590 nm using a Tecan Genios microplate reader (Grödig, Austria). The percentage haemolysis for each sample was calculated using the equation [21]:

2 $$ \mathrm{Percentage}\ \mathrm{haemolysis}=\frac{\kern0.75em \mathrm{Abs}.\mathrm{of}\ \mathrm{sample}-\mathrm{Abs}.\mathrm{of}\ \mathrm{control}\kern1em \times 100}{\mathrm{Abs}.\mathrm{of}\ \mathrm{maximal}\ \mathrm{lysis}-\mathrm{Abs}.\mathrm{of}\ \mathrm{control}} $$

#### Determination of toxicity using mouse peritoneal cell

**Fig. 1:**
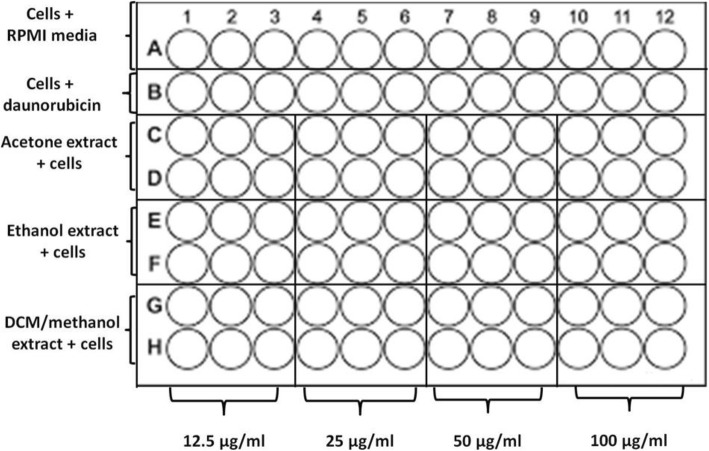
Plate set up for the MTT assay using mouse peritoneal cells, exposed to different solvent extracts from *T. welwitschii* leaves. Cells in RPMI row was the negative control, while the cells daunorubicin row was the positive controls

This work on animals was conducted in accordance with the internationally accepted principles for the protection of animals used for scientific purposes [22]. Six weeks old male laboratory-bred strain of the house mice (BALB/c) of 20–25 g weight were collected from the Animal House at the University of Zimbabwe (Harare, Zimbabwe) and used. The research was carried out according to the rules governing the use of laboratory animals and the experimental protocol was approved by the Faculty of Higher Degrees Committee, Harare, Zimbabwe (HD/71/16). To increase the number of peritoneal cells within the mice, 20% sterile starch solution was intraperitoneally introduced into the mice using a syringe with a 27 g needle. The mice were left for 48 h in plastic cages with unlimited access to food and water in order to allow peritoneal cell yield increase. Total peritoneal cells were isolated as described by Ray and Dittle [23]. Each mouse was euthanized by cervical dislocation. Then sprayed with 70% ethanol and mounted on a styrofoam block on its back. Scissors and forceps were used to cut the outer skin of the peritoneum to expose the inner skin lining the peritoneal cavity. A volume of 5 ml of ice cold PBS with 3% FCS was introduced into the peritoneal cavity using a 27 g needle. Due care was taken to avoid puncturing of organs. After injection, the peritoneum was gently massaged to remove any attached cells into the PBS solution. A 25 g needle, attached to a 5 ml syringe was used to collect the fluid from the peritoneum into tubes kept on ice after removing the needle from the syringe. The cell suspension collected was spurn at 1500 r.p.m. for 10 min in a Rotofix 32A centrifuge. The supernatant was discarded and cells resuspended the cells in RPMI. Cells were cultured in RPMI medium supplemented with 10% Fetal bovine serum (FBS) and 1% PNS (penicillin, neomycin and streptomycin) and incubated in a Shellab incubator (CO_2_ series Sheldon Mfg. Inc., Cornelius, USA) at 37 °C in a controlled atmosphere with 5% CO_2_ for 24 h. Cells were stained with 0.4% trypan blue and viable cells counted using a haemocytometer counting chamber under a Celestron digital light microscope (Celestron, Los-Angeles, USA) using the × 10 objective lens. Toxicity was determined using the MTT assay as described by Mapfunde et al., [24]. Extracts were dissolved in DMSO. Each of the three extracts was double diluted to give concentrations of 12.5, 25, 50 and 100 μg/ml. The final concentration of DMSO in each well was 1%. A typical plate set up is as shown in Fig. 1. The cells were incubated in 96-well plates in the presence of extracts for 24 h at 37 °C in a 5% CO_2_ Shel lab incubator. Each well contained 100 μl of the test substance and 100 μl of 0.5 × 10^5^ cells/ml in RPMI. Cells exposed to the standard anticancer drug daunorubicin (10 μg/ml) were used as the positive control. Cells in RPMI were used as the negative control. After the 24 h incubation, 25 μl of MTT (3-(4, 5-dimethylthiazol-2-yl)-2, 5-diphenyltetrazolium bromide) was added to each well and plates were incubated for 4 h. A volume of 50 μl of DMSO was added and the absorbance of the contents in wells was measured at 590 nm using a Tecan Genios-Pro microplate reader (Tecan Group Ltd. Mӓnnedorf, Switzerland).

#### Statistical analyses

One-way analysis of variance test (ANOVA) with Dunnett’s Multiple Comparison Post Test was used to analyse the results. All sets of data were compared to the control. The values with a *p*-value < 0.05 were considered statistically significant. Graphical and Statistical analyses were carried out using GraphPad Prism 5® Software (Version 5.0, GraphPad Software Inc., San Diego, USA).

## Results

### Yield of extracts

The percentage yield for the various extracts was calculated using the formula:

$$ \mathrm{Percentage}\ \mathrm{yield}\ \left(\%\right)=\frac{\mathrm{Mass}\ \mathrm{of}\ \mathrm{extract}\ \mathrm{obtained}\ \left(\mathrm{g}\right)\times 100}{\mathrm{Mass}\ \mathrm{of}\ \mathrm{plant}\ \mathrm{powder}\ \mathrm{used}\ \left(\mathrm{g}\right)} $$

The results of the percentage yields of the extracts are shown in Table 1.

**Table 1:** Yield of extracts from leaves of *T*. *welwitschii*

Solvent used for extraction	Yield (%)
DCM: methanol	8.06
Hexane	1.66
DCM	0.98
Ethyl acetate	0.90
Acetone	2.53
Methanol	2.53
Ethanol	3.42
Water	0.52

The solvent mixture of DCM: methanol extracted the highest percentage of extracts (8.06%) while water extracted the least percentage of extracts (0.52%). The polar solvents which included acetone, methanol and ethanol yielded greater than 2% extract with water being an exception. The non-polar solvents which included hexane, DCM and ethyl acetate gave yields of less than 2%.

### Antibacterial activities of extracts

**Fig. 2:**
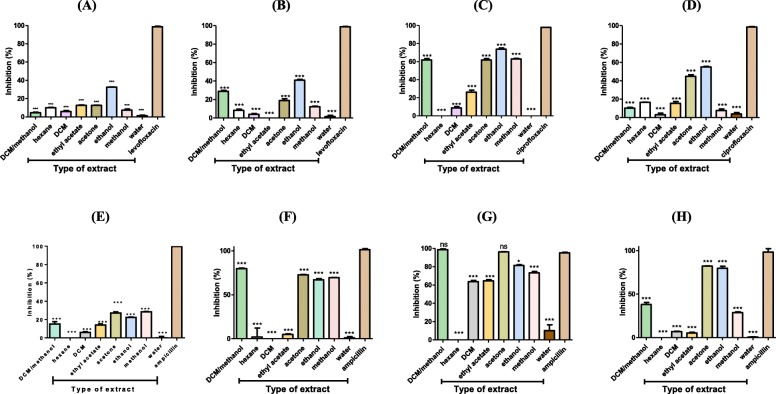
Percentage inhibition of bacterial cells upon treatment with 100 μg/ml extract from leaves of *T*. *welwitschii*. **a** *K. pneumoniae*, **b** *S. pneumoniae*, **c** *S. pyogenes*, **d** *B. subtilis*, **e** ATCC strain of *S. aureus*, **f** clinical strain of *S. aureus*, **g** ATCC strain of *P. aeruginosa*, and **h** clinical strain of *P. aeruginosa*. Values are for mean ± standard deviation (error bar) for *n* = 4. The asterisks indicate a significant difference from the control with *p < 0.05, ***p < 0.001 and ns mean no significant difference

The percentage inhibition of bacterial growth caused by leaf extracts of a 100 μg/ml concentration is as shown in Fig. 2. All leaf extracts showed varied antibacterial activities against test bacteria. The most significant growth inhibition by the extracts was against *P. aeruginosa* ATCC compared to the other seven bacterial isolates. The growth inhibition of the clinical strain of *P. aeruginosa* by most of the extracts was lower than that in the ATCC strain *P. aeruginosa*. The extracts exhibited the least growth inhibition against the clinical strain of *K. pneumoniae* compared to the rest of the test isolates. Of the eight extracts, the acetone, ethanol and DCM/methanol leaf extracts showed growth inhibitory activities of 96, 81 and 99% respectively against *P. aeruginosa* ATCC. Polar extracts (acetone, ethanol and methanol) with the exception of the water extract showed growth inhibition of greater than 60% against *P. aeruginosa* ATCC. The non-polar extracts; ethyl acetate, DCM and hexane showed less than 60% inhibition of the growth of *P. aeruginosa* ATCC. The ethanol, acetone and DCM/ methanol leaf extracts were used in subsequent biochemical and toxicity tests since they had shown higher growth inhibition of *P. aeruginosa* ATCC in comparison to the other extracts. Having noted that growth inhibition by the extracts was greatest against the ATCC strain of *P. aeruginosa* compared to the rest of the test isolates, subsequent biochemical and toxicity tests were performed using this strain. Total inhibition of bacterial growth by the standard antibiotics were at concentrations of: 50 μg/ml for the ATCC strain of *P. aeruginosa*; 25 μg/ml for the clinical strain of *P. aeruginosa*; 0.4 μg/ml for the ATCC and clinical strain of *S. aureus*; 0.5 μg/ml for *S. pyogenes* and *B. subtilis*; 0.25 μg/ml for *S. pneumoniae*.

### Effects of extracts on efflux activity

**Fig. 3:**
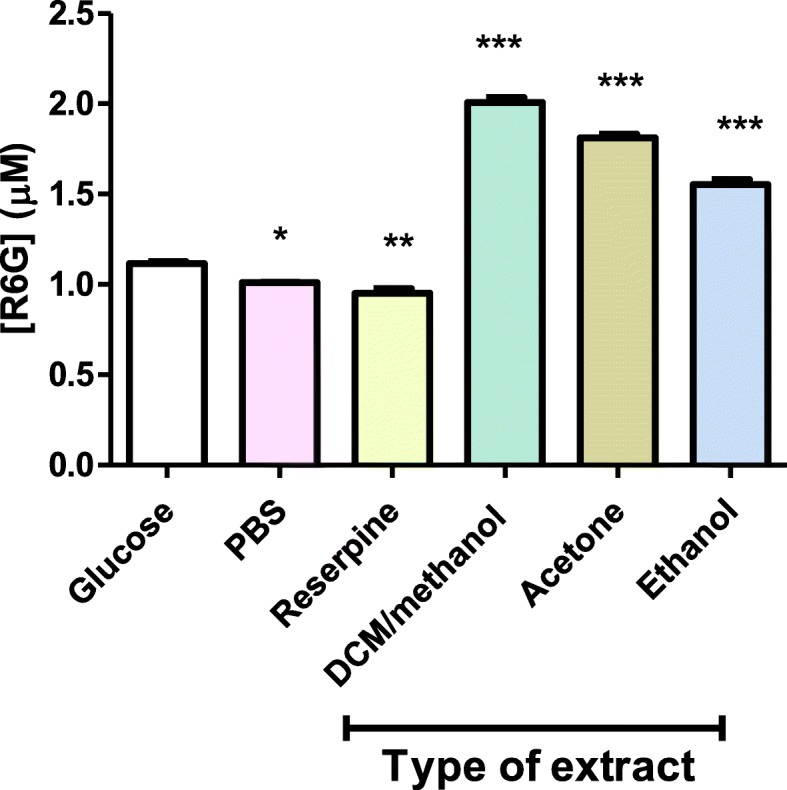
The effects the acetone, ethanol and DCM/methanol leaf extracts from *T*. *welwitschii* on efflux pump activity of the ATCC strain of *P. aeruginosa*. Cells exposed to glucose served as the positive control where active efflux occurred maximally. The error bars show the standard deviation from the mean of two samples read twice. The asterisks indicate a significant difference from the control with *p < 0.05, **p < 0.01 and ***p < 0.001

The amount of R6G extruded in the presence acetone, ethanol or DCM/methanol extracts was used to assess the effects of the extracts on efflux pump activities in *P. aeruginosa* (Fig. 3). The presence of the ethanol, acetone and DCM/methanol leaf extracts stimulated the extrusion of R6G from the bacterial cells. The cells exposed to glucose showed higher efflux of R6G when compared to cells exposed to the efflux pump inhibitor (EPI) reserpine. The presence of the acetone, ethanol and DCM/methanol extracts resulted in an increased extrusion of R6G from *P. aeruginosa* compared with the extrusion in the presence of reserpine or glucose.

### Membrane damage potential of extracts

**Fig. 4:**
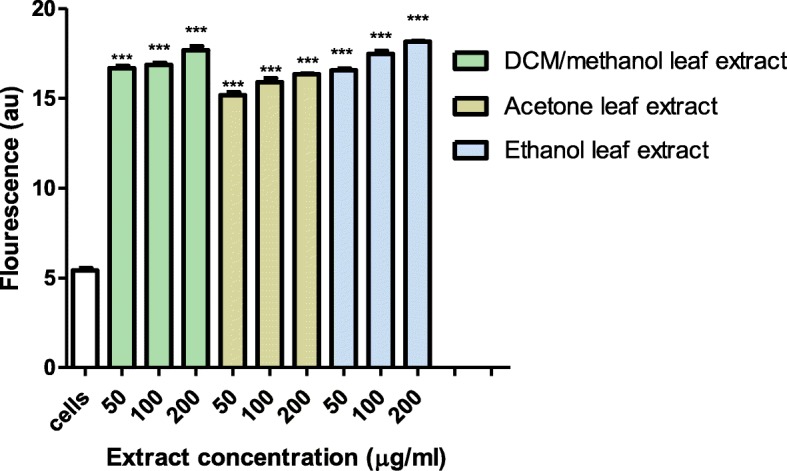
Fluorescence of propidium iodide bound to nucleic acids of *P. aeruginosa* cells after exposure to the acetone, ethanol and DCM/methanol leaf extracts from *Triumfetta welwitschii*. Cells with no extract were used as the control. Values are for mean ± standard deviation (error bar) for *n* = 3. The asterisks indicate a significant difference from the control with ***p < 0.001

Effects of the DCM/methanol, acetone and ethanol leaf extracts on bacterial membrane integrity was determined by exposing *P. aeruginosa* to varying concentrations of the extracts followed by staining with propidium iodide. The exposure to leaf extracts resulted in bacterial cell membrane disruption. Membrane disruption was evidenced by an increased uptake of propidium iodide by the exposed cells in comparison to the unexposed cells (*P* < 0.05) (Fig. 4). All three extracts were able to cause significant membrane permeability resulting in nucleic acid leakage from *P. aeruginosa* cells when compared to the control. The highest amount of nucleic acid leakage was observed in cells exposed to 200 μg/ml ethanol leaf extract while the acetone leaf extract caused the least nucleic acid leakage at the same concentration.

### Effects of extracts on sheep erythrocytes

**Fig. 5:**
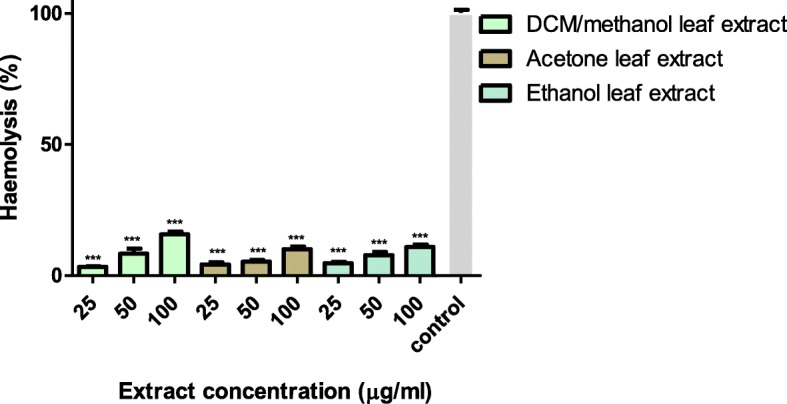
The percentage haemolysis of sheep erythrocytes induced by exposure to different concentrations of the DCM/methanol, acetone and ethanol leaf extracts from *T. welwitschii*. Cells with no extract were used as the control. Values are for mean ± standard deviation (error bar) for *n* = 3. The asterisks indicate a significant difference from the control with ***p < 0.001

The haemolysis of sheep erythrocytes induced by the acetone, ethanol and DCM/methanol leaf extracts from *T*. *welwitschii* expressed as a percentage is as shown in Fig. 5. At a concentration of 100 μg/ml, the DCM/methanol leaf extract showed the highest haemolytic effect when compared to the ethanol and acetone extracts. All three extracts showed a dose-dependent haemolytic effect against the sheep erythrocytes. The DCM/methanol leaf extracts showed haemolytic activity of 16%. The acetone and ethanol leaf extracts at a concentration of 100 μg/ml had haemolytic activity of 10 and 11% respectively.

### Effects of leaf extracts on mouse peritoneal cells

**Fig. 6:**
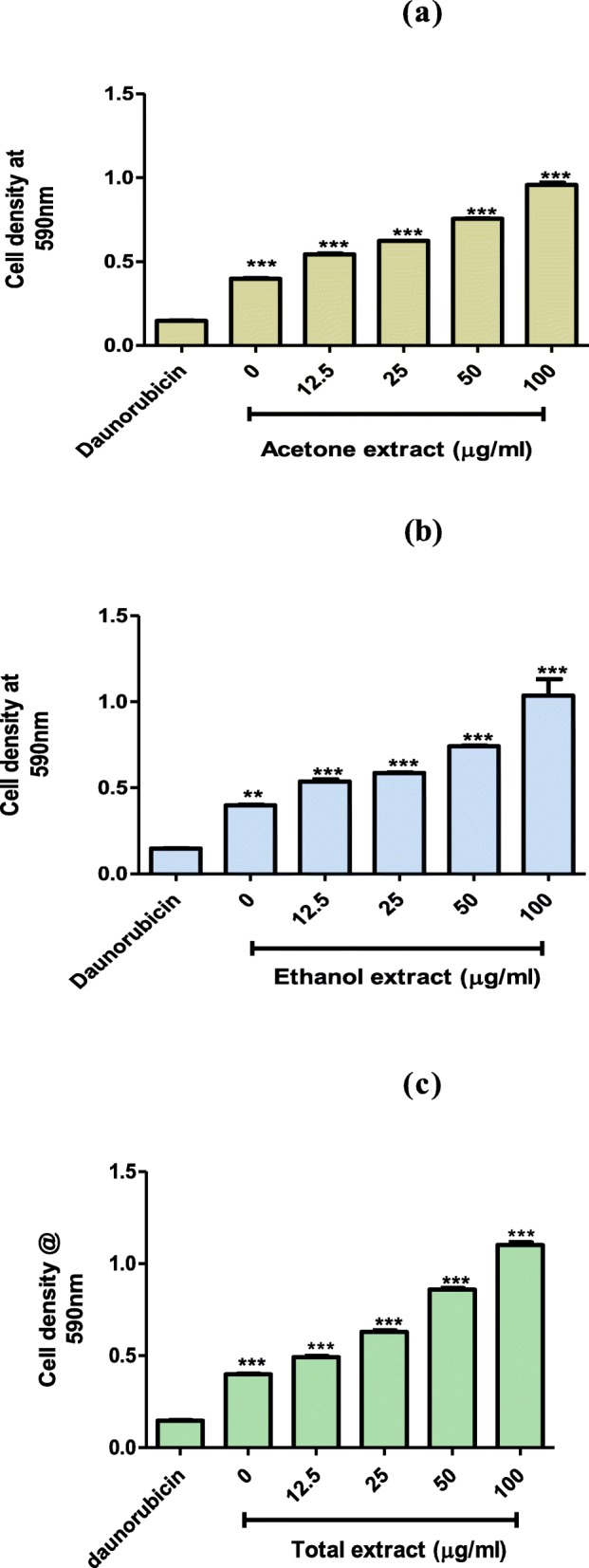
The effects of the **a** acetone, **b** ethanol and **c** DCM/methanol leaf extracts of *T. welwitschii* on mouse peritoneal cells. Cells with daunorubicin a standard antibiotic were used as the control. Values are for mean ± standard deviation (error bar) for *n* = 3. The asterisks indicate a significant difference from the control with **p < 0.01 and ***p < 0.001

Toxicity of the acetone, ethanol and DCM/methanol leaf extracts from *T. welwitschii* was tested on mouse peritoneal cells. The effects of the extracts on the growth of mouse peritoneal cells are as shown in Fig. 6. All test samples were non-toxic to the mouse peritoneal cells. The three extracts showed a dose-dependent increase in mouse peritoneal cells proliferation. The DCM/methanol leaf extract and the ethanol leaf extracts had the highest and least proliferation stimulatory properties respectively.

## Discussion

The search for new antimicrobials is frequently based on ethnobotany and ethnopharmacology [25]. *T*. *welwitschii* was selected based on its ethnomedicinal use in the Southern parts of Africa [5, 6]. Since work had already been done on the roots [7–9] this study focused on the leaves of the plant as there is a knowledge gap pertaining the pharmacological value of the leaves of the plant. Solvents of varying polarities were used to prepare extracts from leaves of *T*. *welwitschii*. Different solvents extract different phytochemical groups; therefore, serial exhaustive extraction was used to enhance the isolation of phytochemicals from the complex crude mixture [26]. The DCM: methanol solvent mixture gave the highest percentage yield (8.06%). The solvent mixture constitute of a polar and non-polar solvent which must have facilitated the extraction of both polar and non-polar phytochemicals. Polar solvents with the exception of water gave yields of more than 2% while non-polar solvents gave yields of less than 2%. Martini and Eloff [27] showed that the polar solvents have higher extracting potential than the non-polar solvents.

Leaf extracts from *T. welwitschii* possessed varying potential of antibacterial activity against *P. aeruginosa*, *S. aureus*, *K. pneumoniae*, *S. pneumoniae*, *S. pyogenes* and *B. subtilis* (Fig. 2)*.* Of the eight test isolate, *P. aeruginosa* ATCC was the most inhibited by the majority of extracts. It is worth noting that the Gram-negative *P. aeruginosa* was inhibited by most of the extracts more than the Gram-positives *S. aureus*, *B. subtilis*, *S. pneumoniae* and *S. pyogenes*. Gram negatives possess two cellular membranes, with the outer membrane covered with lipopolysaccharides, making it a formidable barrier for molecules to penetrate [28] which deviates from the expected results. In this study, the disruption of membrane integrity was shown to be the mode of action of the three extracts. The penetration of the outer membrane of the Gram-negative *P. aeruginosa* by the extracts may have been achieved through the pre-disruption of the membrane.

The acetone, ethanol and DCM: methanol leaf extracts from *T. welwitschii* were the most active extracts against the ATCC strain of *P. aeruginosa*. Acetone, ethanol and methanol (in the DCM: methanol mixture) are polar solvents known to extract a wide range of phytochemicals [27]. Antibacterial activities shown by these extracts may be attributed to phenols, flavonoids [29] and coumarins [30] the common secondary metabolites in *T*. *welwitschii*. A total of six and two extracts showed more than 50% growth inhibition against the ATCC and clinical strains of *P. aeruginosa* respectively. The inhibition of growth of the clinical strain of *P. aeruginosa* by most of the extracts was lower compared to that of the ATCC strain. Laboratory strains have been sub-cultured for years since they were first isolated. A diversity of genotypes subsequently changes over time [31] hence the different responses noted for the clinical and laboratory strains. These findings on the antibacterial activity of extracts from *T. welwitschii* plant make the plant a possible source of compounds to explore for novel lead compounds for drug development against *P. aeruginosa*.

A wide range of mechanisms provide bacteria with resistance to antibiotics; these include target-site modification and antibiotic inactivation among others. The expression of efflux pumps by some human pathogenic bacteria confers multidrug resistance (MDR). A single pump may provide bacteria with resistance to an extensive range of chemically and structurally different compounds. Natural products are a possible source of efflux pump inhibitors [32–34]. The R6G efflux assay was carried out to determine the potential use of the acetone, ethanol and DCM/methanol leaf extracts from *T*. *welwitschii* as efflux pump inhibitors. The R6G assay involves preloading the cell with a fluorescent substrate (R6G) prior to the efflux assay. After the loading step, R6G accumulates within the cells. Cells are then washed to remove R6G on the outer surface of cells. Subsequently, glucose is added to the culture as a source of energy, and the efflux of R6G is measured by fluorimetry [35]. A known EPI (e.g reserpine) is included as a positive control for inhibition of the efflux of R6G. Results from the R6G efflux (Fig. 3) showed that there was increased efflux of R6G in the presence of plant extracts compared to cells in glucose. The plant extracts stimulated efflux. Thus, the extracts used in this study lacked efflux pump inhibitory activity. While inhibition of efflux pumps seems to be a worthy approach for improving the efficacy of antibiotics which are substrates of such pumps, it is important to identify antibiotics and target bacteria for which this approach would be the most applicable [36].

Antibacterial agents, usually act on the membranes of bacteria by causing disruption and permeabilisation [37]. The antibacterial mode of action of the acetone, ethanol and DCM: methanol leaf extracts from *T*. *welwitschii* on the membrane integrity of *P. aeruginosa* was determined using propidium iodide a fluorescent nucleic acid stain. Live bacterial cells are impermeable to propidium iodide, but upon membrane disruption or permeabilisation, propidium iodide can enter the cells [18]. The exposure of *P. aeruginosa* to the three leaf extracts resulted in bacterial cell membrane disruption as evident from the increased uptake of propidium iodide in comparison to the unexposed cells (Fig. 4). The increased fluorescence of propidium iodide by cells showed that there was disruption of the cell membrane since propidium iodide exclusively bind to nucleic acids of dead cells with damaged membranes only and not live cells. It has been reported in other studies that some extracts cause membrane damage leading to nucleic acid leakage [38], and induce cell damage [39]. Among extracts that cause membrane damage causing leakage of cell materials can be found also the *Plumbago zeylanica* root [37], *Trianthema portulacastrum* leaf [40], and *Ocimum basilicum* [41].

For a plant extract to be useful, it has to possess bioactive properties and exhibit non-cytotoxic profile. Some plants possessing bio-active components may show toxicity thus it is important to investigate the primary toxicity of plant extracts. Several researchers have used erythrocytes as a model system for determining the interaction of drugs with mammalian membranes [42–45]. The erythrocyte model has been commonly used in toxicity profiling as it provides a direct indication of toxicity of injectable preparations in addition to a general indication of membrane toxicity [46]. Haemolysis is a result of the destruction of the erythrocyte caused by the lysis of the membrane lipid bilayer. The lysis of erythrocytes can cause anaemia, an increase in plasma haemoglobin leading to nephrotoxicity and vasomotor instability [47]. In the haemolytic assay, when the erythrocyte suspension was diluted in Drabkin’s, the reagent haemolysed the erythrocytes. The haemolysis released haemoglobin into the solution. The Fe^2+^ of the haemoglobin molecules were oxidised by potassium ferricyanide to Fe^3+^. This oxidation resulted in the formation of methaemoglobin which combined with the cyanide ions to form cyanmethemoglobin, a stable compound colour pigment read calorimetrically at 590 nm [48]. The acetone, ethanol and DCM: methanol leaf extracts from *T*. *welwitschii* showed haemolytic activity of 10–16% (Fig. 5). According to Vidhya and Udayakumar [49], a 10–49% haemolytic activity is rated as slightly toxic. Therefore, the 10–16% haemolytic activity obtained for the three leaf extracts from *T*. *welwistchii* is an indicator of non-significant toxicity to erythrocyte membrane, consequently favouring further study of the plant species.

Macrophages are highly phagocytic and considered to be essential immune effector cells that participate in innate and adaptive immune responses. Since the functioning of macrophages can be altered depending on their surrounding environment and the stimuli they are exposed to [50], they were used as a typical model to study the cytotoxicity of plant extracts. The potential of plant extracts to inhibit the growth or viability of murine macrophages can, therefore, be used as an indication of toxicity. Viability of mouse peritoneal cell was determined using the MTT assay. The yellow tetrazolium MTT salt was reduced by metabolically active cells by the action of dehydrogenase enzymes giving a purple colour. The intensity of the purple colour was used to calorimetrically measure viable cells [51]. The results of the mouse peritoneal cells exposed to the acetone, ethanol and DCM: methanol extracts from *T*. *welwistchii* (Fig. 6) showed that cell survival increased with increasing extract concentration. The proliferative effect of the three extracts on the mouse peritoneal cells was an indication that the leaf extracts were not toxic towards the mouse peritoneal cells. Similar results were reported by Ragupathi., et al [52], saponins isolated from *Quillaja saponaria* tree bark stimulated the production of immune cells. Sun et al., [53], showed that most plant polypeptides promote the proliferation of macrophages among other immune cells. Therefore, the results of this study provide evidence that the acetone, ethanol and DCM/methanol leaf extracts are not toxic to mouse peritoneal cells but may stimulate their growth. The extracts may boost growth of the immune cells which are vital in fighting some bacterial infections.

## Conclusion

The acetone, ethanol and total leaf extracts from *T*. *welwistchii* showed antibacterial activity against *P. aeruginosa* ATCC. Membrane disruption was the mode of action against the bacteria for the three extracts. The three leaf extracts showed low toxicity, thus, they could be potential sources of alternative antimicrobials against infections caused by *P. aeruginosa*. Studies will be conducted on the extracts in order to isolate and characterise the specific compounds responsible for these antibacterial activities.

## Data Availability

The data sets generated during and/ analysed during the current study are available from the corresponding author on reasonable request.

## Abbreviations

ANOVA: One way analysis of variance
ATCC: American type control culture
CFU: Colony forming units
DCM: Dichloromethane
DMSO: Dimethyl sulfoxide
MTT: 3-(4,5-dimethylthiazolyl)-2,5-diphenyltetrazolium
PBS: Phosphate buffered saline
R6G: Rhodamine 6G
RPMI: Roswell Park Memorial Institute
TSA: Tryptic soy agar
TSB: Tryptic soy broth
